# Is There an Opportunity for Current Chemotherapeutics to Up-regulate MIC-A/B Ligands?

**DOI:** 10.3389/fphar.2017.00732

**Published:** 2017-10-17

**Authors:** Kendel Quirk, Shanmugasundaram Ganapathy-Kanniappan

**Affiliations:** Division of Interventional Radiology, Russell H. Morgan Department of Radiology and Radiological Science, Johns Hopkins University School of Medicine, Baltimore, MD, United States

**Keywords:** cancer, natural killer cells, doxorubicin, immunotherapy, MIC-A/B

## Abstract

Natural killer (NK) cells are critical effectors of the immune system. NK cells recognize unhealthy cells by specific ligands [e.g., MHC- class I chain related protein A or B (MIC-A/B)] for further elimination by cytotoxicity. Paradoxically, cancer cells down-regulate MIC-A/B and evade NK cell’s anticancer activity. Recent data indicate that cellular-stress induces MIC-A/B, leading to enhanced sensitivity of cancer cells to NK cell-mediated cytotoxicity. In this *Perspective* article, we hypothesize that current chemotherapeutics at sub-lethal, non-toxic dose may promote cellular-stress and up-regulate the expression of MIC-A/B ligands to augment cancer’s sensitivity to NK cell-mediated cytotoxicity. Preliminary data from two human breast cancer cell lines, MDA-MB-231 and T47D treated with clinically relevant therapeutics such as doxorubicin, paclitaxel and methotrexate support the hypothesis. The goal of this *Perspective* is to underscore the prospects of current chemotherapeutics in NK cell immunotherapy, and discuss potential challenges and opportunities to improve cancer therapy.

## Introduction

Cancer continues to be one of the leading causes of death (Global Burden of Disease Cancer Collaboration et al., 2015). Our understanding of carcinogenesis has significantly advanced in the recent decades. Consequently, several novel strategies and potential anticancer therapeutics have emerged, although with limited success in translation. Some of the common challenges that block successful clinical translation of potential therapeutics include resistance to therapy, metastasis, etc. Apart from the biological challenges, the cancer drug development program is also impeded by the high-cost and extensive time incurred for the development of *de novo* drugs (Ishida et al., 2016). Recently, there has been an interest to exploit serendipitous anticancer effects of therapeutics that are indicated for other ailments. This process of recognition of new indications of a clinically approved therapeutic is referred as “drug repositioning” or “drug repurposing” (Ishida et al., 2016). Emerging reports indicate that such drug repositioning and repurposing could have desirable outcome in the management of cancer. For example, compounds of cardiovascular treatments (Ishida et al., 2016), anti-diabetic agents (Gadducci et al., 2016) and HIV therapeutics (Maksimovic-Ivanic et al., 2017) have been found to promote anticancer effects. In this context, extended application of current chemotherapeutics to enhance the efficacy of immunotherapy has also been indicated (Fournier et al., 2017).

**FIGURE 1:**
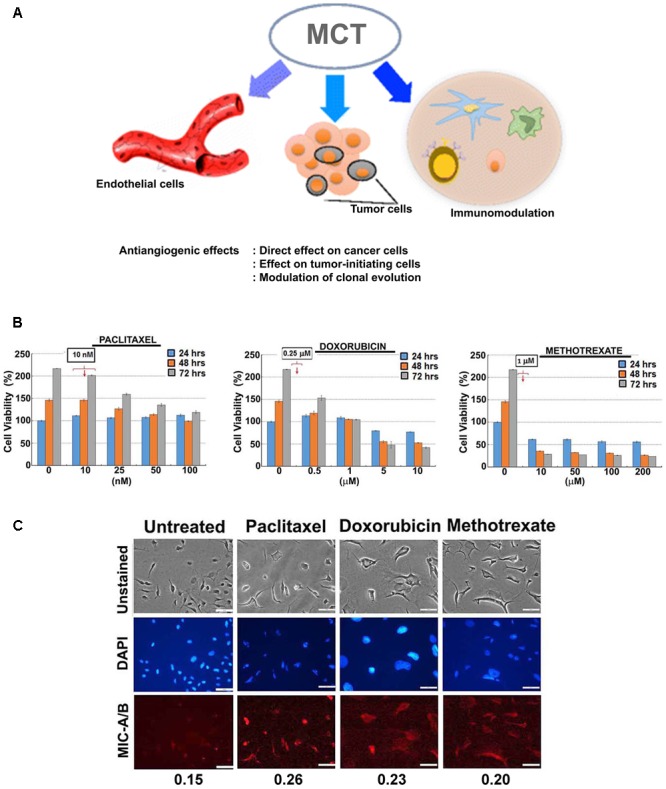
Effect of sub-lethal non-toxic dose of chemotherapeutics on MIC-A/B induction in MDA-MB-231 cells. **(A)** Schematic showing potential effects of metronomic chemotherapy (MCT) (e.g., angiogenic inhibitor) on cancer and immune modulation [reproduced with permission of Springer, aaa Springer Science+Business Media New York 2016 (Romiti et al., 2017)]. **(B)** Determination of sub-lethal, non-toxic dose of respective chemotherapeutics over 24, 48 and 72 h of treatment. The concentrations indicated in the square box is the dose used for metronomic treatment. **(C)** Effect of respective chemotherapeutics on the induction of MIC-A/B as evidenced by specific staining (red fluorescence). The nuclear stain by DAPI (blue) and light microscopic images have been shown to indicate cell-specific staining of MIC-A/B (red fluorescence). Numerical data below the fluorescent images represent specific-signal intensity obtained by the ratio between DAPI and MIC-A/B staining. Scale-100 μm.

Cancer chemotherapeutics at their maximum tolerated dose or the most efficacious dose have long been known to cause undesirable effects, including immune-suppression (Hersh and Oppenheim, 1967). Reports from two independent groups, Browder et al. (2000) and Klement et al. (2000) demonstrated that repeated, low-dose chemotherapy at frequent cycles promote desirable anticancer effects. Interestingly, a decade earlier it was shown that a combinatorial approach using a low-dose of cyclophosphamide with a low-dose of IL-2 had synergistic, improved anticancer effects (Eggermont and Sugarbaker, 1988). However, the inferences were mainly focused on the combination therapy. Nonetheless, these studies provided the foundation for the modern concept of “metronomic therapy.” Consequently, metronomic treatment has gained much attention (**Figure 1A**) (Romiti et al., 2017), and has been expected to play a significant role in the context of personalized medicine as well (Andre et al., 2014). Concomitantly, data also emerged indicating that conventional maximum tolerated dose of chemotherapeutics affect anticancer immune cells (e.g., NK cells) (Saijo et al., 1982; Sewell et al., 1993). Furthermore, post-chemotherapy though a recovery in total number of immune cells was observed, the functional recovery was not evident indicating loss of immune cell function in breast cancer as well as lung cancer (Saijo et al., 1982; Sewell et al., 1993). On one hand, the anticancer function of immune cells such as NK cells has been known to be affected by high dose chemotherapeutics; on the other hand, low-dose metronomic therapy improves anticancer effects. With this background, emerging concepts point to the optimization of drug regimen that could augment or facilitate anticancer immune activity (Emens et al., 2001; Emens and Middleton, 2015). Yet, there is paucity of data on the immunotherapeutic potential of chemotherapeutics to enhance the efficacy and/or opportunity for natural killer (NK)-cells, a principal component of the immune system. Here, in this *Perspective* in the light of recent research, we discuss the potential of sub-lethal, non-toxic dose of current chemotherapeutics to induce the expression of MIC-A/B to sensitize cancer cells to NK-cell mediated cytotoxicity.

## Tumor Cells, Immune Evasion, And NK Cells

Cancer cells evade immune surveillance, and this “immune evasion” has recently been recognized as one of the hallmarks of cancer (Hanahan and Weinberg, 2011). Though earliest report on the anticancer potential of the immune system dates back to the 19th century (Coley, 1898), only in the past few decades the clinical relevance and plausible outcomes of immunotherapies have been recognized (Burnet, 1957). For example, recent reports on tumor microenvironment (TME) and understanding the impact of cancer metabolism on TME have shed light in deciphering the anti-immune properties of TME (Ganapathy-Kanniappan, 2017a,b). Emerging data indicate that the alteration of TME could impact the antitumor immune response (Husain et al., 2013; Fu et al., 2015). Among immune cells, besides the T cells, studies on NK cells have also gained momentum. NK cells are an integral component of the immune system and are considered as the “first line” of defense (Lodoen and Lanier, 2006). NK cells detect and target unhealthy or diseased cells including cancer, and induce cytotoxicity. Thus, NK cell-mediated cytotoxicity is an effective anticancer immunotherapeutic approach (Ljunggren and Malmberg, 2007). Mechanistically, cell surface receptors on NK cells recognize specific ligands (commonly known as NK-G2D ligands) on target cells to induce cytolytic processes. Among the NKG2D ligands, MHC- class I chain related protein A or B (MIC-A/B) are known to be up-regulated during cellular pathology. Paradoxically, cancer cells have been known to reduce the surface expression of MIC-A/B ligands through multiple mechanisms such as cleavage/shedding of the extracellular domain of MIC-A/B or down-regulation of expression (Rzymski et al., 2012; Chitadze et al., 2013). Multiple lines of evidence indicate that restoration of MIC-A/B expression render cancer cells sensitive to NK cell mediated cytotoxicity (de Kruijf et al., 2012; Okita et al., 2012). Thus, interference with cancer’s mechanism of down-regulation of MIC-A/B to up-regulate the expression may be an effective approach to enhance cancer’s sensitivity to NK cells. Akin to this, disruption of energy metabolism of cancer (Fu et al., 2015), induction of thermal stress (Dayanc et al., 2013), exposure to pro-oxidants such as hydrogen peroxide (Yamamoto et al., 2001) and reactive oxygen species (Soriani et al., 2014) have been shown to up-regulate the expression of MIC-A/B in cancer. However, the underlying mechanism of such cellular or metabolic stress-related MIC-A/B up-regulation remains to be known. Nevertheless, the characteristic feature that MIC-A/B are stress-inducible provides a window of opportunity to envisage clinically relevant approaches to induce stress in cancer cells.

## Chemotherapeutics and the Potential for Sensitization to NK Cells

Current chemotherapeutics play a pivotal role in the management of cancer, especially in advanced stages such as metastatic cancers. Ever since the recognition of chemical agents as potential therapeutics in the dawn of 20th century, the field of chemotherapy has advanced remarkably (refer review by DeVita and Chu, 2008). While some chemotherapeutics have been indicated to interfere with the efficacy of NK cell mediated killing of cancer cells others have proven to be effective in enhancing the outcome of NK cell mediated immunotherapy. Besides, in the presence of natural compounds (e.g., fruit extracts of *Morus alba* L.) chemotherapeutic like 5-fluorouracil demonstrated increased anticancer efficacy which involved enhanced NK cell activity (Markasz et al., 2007). Similarly, inhibitors of histone deacetylases (HDACs) such as Trichostatin A have been shown to sensitize cancer cells to NK cell mediated cytotoxicity (Tiper and Webb, 2016; Shin et al., 2017). Recently, Yang et al. (2015) have documented that the HDAC inhibitor, suberoylanilide hydroxamic acid (SAHA) upregulates MIC-A/B by facilitating gene-specific acetylation. Thus, deregulation of epigenetic mechanisms have been indicated to up-regulate MIC-A/B based on genetic regulation.

Irrespective of the diverse class of chemotherapeutics such as DNA-damaging agents (e.g., doxorubicin), antimetabolites (e.g., methotrexate), mitotic inhibitors (e.g., paclitaxel), nucleotide analogs (6-mercaptopurine) or inhibitors of topoisomerases (e.g., etoposide), anticancer agents in general mediate their effects by induction of cell death mechanisms (Herr and Debatin, 2001). Noteworthy, a common underlying mechanism is the induction of specific or overall cellular stress, and the severity of which determines the outcome (i.e.) cell death (Herr and Debatin, 2001). Invariably, the majority of chemotherapeutics implicate the induction of cellular-stress during their anticancer effects (Gewirtz, 1999; Minotti et al., 2004). Since chemotherapeutics could induce cellular-stress and the MIC-A/B ligands required for NK cell recognition are stress-inducible, it is intriguing to verify whether chemotherapeutics could be exploited to up-regulate MIC-A/B. To test this hypothesis it is imperative to include couple of guidelines. (i) The objective of using the chemotherapeutic is not to achieve cytotoxicity but to induce the expression of MIC-A/B to facilitate NK cell mediated cytotoxicity. This would facilitate effective infiltration and targeting of cancer cells by the NK cell population providing a repertoire of immunological responses against cancer. (ii) The selection of chemotherapeutic dose (sub-lethal, non-toxic low-dose) should be sufficient to cause sustainable cellular stress to allow the induction of MIC-A/B.

**FIGURE 2:**
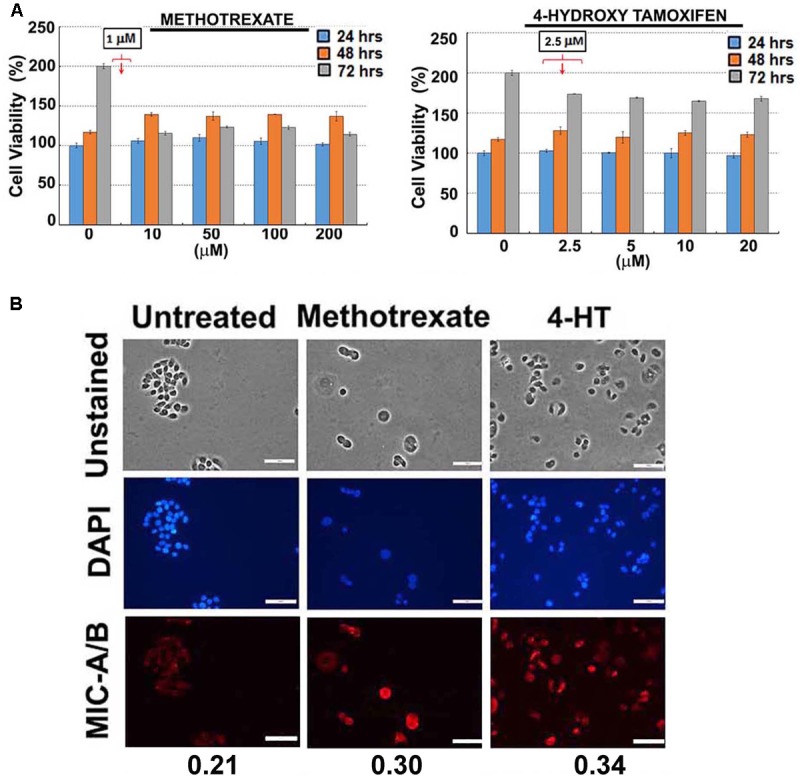
Effect of sub-lethal non-toxic dose of chemotherapeutics on MIC-A/B induction in T47D cells. **(A)** Determination of sub-lethal, non-toxic dose of respective chemotherapeutic over 24, 48 and 72 h of treatment. The concentrations indicated in the square box is the dose used for metronomic treatment. **(B)** Effect of respective chemotherapeutics on the induction of MIC-A/B as evidenced by specific staining (red fluorescence). The nuclear stain by DAPI (blue) and light microscopic images have been shown to indicate cell-specific staining of MIC-A/B (red fluorescence). Numerical data below the fluorescent images represent specific-signal intensity obtained by the ratio between DAPI and MIC-A/B staining. Scale-100 μm.

For preliminary investigation, two human breast cancer cell lines, MDA-MB-231 and T47D were examined with one or more of the following clinically relevant therapeutics such as doxorubicin, paclitaxel, 4-hydroxy tamoxifen (4-HT) and methotrexate. As indicated in **Figures 1B**, **2A**, the sub-lethal, maximum non-toxic dose of respective chemotherapeutics was determined (IC_10_) and the cells were subjected to treatment at the dose equivalent or lesser than the IC_10_. The IC_10_ was determined by Celltiter-Glo Bioluminescent assay (Promega, Co., United States). In brief, a day before the metronomic treatment, cells growing in log-phase were plated to attain ∼60% confluency in 96-well plates (for toxicity assay). The following day, metronomic treatment was initiated with the replacement of complete-growth medium with various concentrations of the drugs to be tested. The drug-containing media was replaced every 48 h, and the viability assay was performed 4-days from the initiation of treatment. For MIC-A/B immunostaining, only the chosen concentration (the dose equivalent or lesser than the IC_10_) was used, but the cells were plated in 8-well chamber/cover-glass slides (for immunofluorescence imaging). The treatment was performed as described and staining was performed with specific antibodies. Immunofluorescence imaging showed that treatment with sub-lethal, non-toxic dose of chemotherapeutics elevated the expression of MIC-A/B compared to untreated (control) cells (**Figures 1C**, **2B**). Quantification of specific signal intensity normalized with nuclear stain (DAPI) signal showed chemotherapy-dependent induction of MIC-A/B (**Figures 1C**, **2B**). As discussed earlier, genetic or epigenetic regulation of MIC-A/B by specific inhibitors like SAHA have already been known (Yang et al., 2015). Yet, the up-regulation of MIC-A/B by clinically relevant chemotherapy-dependent cellular stress remains to be known.

## Opportunities, Challenges, And Future Directions

Clinical data obtained from 30 patients demonstrated that the functional status of NK cells during or after chemotherapy strongly correlated with the disease-free survival or tumor recurrence (Mackay et al., 1983). Similarly, an overall increase in immune-infiltration of tumors following chemotherapy has also been known (Hernberg et al., 1997). However, due to the lack of mechanistic insights, skepticism overruled the immunotherapeutic potential of chemotherapeutics. Emerging reports unravel the possible mechanisms and provide significant insights on chemotherapy-related sensitivity of cancer to immune cells such as T cells and NK cells. Using clinically relevant chemotherapeutics it has also been demonstrated that induction of cellular stress or genotoxic stress render cancer cells sensitive to NK cells (Fine et al., 2010). Further, it has been shown that chemotherapy-dependent down-regulation of C-type lectin related receptor on cancer cells was coordinated with an up-regulation of NKG2D ligands (Fine et al., 2010). Note, MIC-A/B are also NKG2D ligands that are recognized by NK cells. In fact, in end-stage patients it has been demonstrated that low-dose metronomic treatment with cyclophosphamide depletes the regulatory T cells (T_regs_- that inhibit the cytotoxic T lymphocytes), and restores the activity of T-cells as well as NK cells (Ghiringhelli et al., 2007). However, a direct molecular link between tumor sensitivity and NK cell efficacy following chemotherapy still remains obscure. Recent data demonstrated that induction cellular stress (e.g., H_2_O_2_, thermal stress, metabolic stress) followed by the up-regulation of MIC-A/B is a direct molecular link that sensitizes cancer cells to NK cell mediated cytotoxicity (Yamamoto et al., 2001; Dayanc et al., 2013; Fu et al., 2015). It has also been shown that such stress conditions decrease the rate of shedding or cleavage of the MIC-A/B a mechanism that enables cancer cells to evade NK cell recognition (Chitadze et al., 2013). These reports unequivocally indicate that induction of cellular stress could be pivotal to up-regulate NKG2D ligands (Fine et al., 2010) and sensitize cancer cells to NK cell activity.

The preliminary data shown here certainly necessitates detailed investigation for further validation. Yet, the results provide first indication of the possible application of current chemotherapeutics at non-lethal metronomic doses to induce cellular stress followed by the expression of stress-inducible MIC-A/B. Importantly, as the therapeutics are used at very low, non-toxic doses it is likely to avoid or prevent potential systemic toxicities or undesirable effects that are frequently encountered with conventional chemotherapy. For example, chemotherapy-related complications on gastrointestinal tract (Boussios et al., 2012) and cardiovascular toxicities (Swain et al., 2003; Jones et al., 2007; Khouri et al., 2012) have already been reported. Furthermore, chemotherapy related toxicities on the central nervous system (e.g., methotrexate) (Cordelli et al., 2017) and cardiomyopathy (e.g., doxorubicin) have also been reported (Chatterjee et al., 2010). Besides toxicities, the undesirable effect of some therapeutics (e.g., tamoxifen) involves impact on patient’s face, eyelids, and eyebrows, resulting in frequent visits to the optometrist as well (Omoti and Omoti, 2006).

Paclitaxel, doxorubicin, and methotrexate are common chemotherapeutics approved for the use in the treatment of many cancers. However, using the maximum effective dose with extended periods between treatment cycles has proven to decrease the outcome, with increased systemic toxicity. Recently, metronomic chemotherapy has been suggested as an alternative option to mitigate unwanted side-effects of maximum effective dose (Scharovsky et al., 2009). Thus, by using non-toxic, sub-lethal dose the risk of systemic toxicity is likely to be lowered, if not eliminated. More importantly, such low-dose chemotherapeutics would not hinder or block host immune cells’ function.

Arguably, the use of low-dose chemotherapeutics by metronomic treatment may contribute for the emergence of a resistant or “addiction” phenotype. Such cancer cells may become insensitive to any dose escalation if necessary. In principle, cancer cells that are subjected to cellular stress and induction of MIC-A/B would be sensitive to NK cells hence would be eliminated. Thus, cells that are exposed to low-dose metronomic treatment are likely to be eliminated by NK cell mediated cytotoxicity. Furthermore, data also indicate that cancer cells that acquired resistance to low-dose chemotherapy are still sensitive to the maximum tolerated effective dose (Emmenegger et al., 2011). So, it is plausible that despite the low-dose exposure the cancer cells still be responsive to high-dose chemotherapy. Nevertheless, additional pre-clinical as well as clinical investigations are mandatory to verify any potential concerns. Future studies on the stability and half-life of MIC-A/B ligands that are induced by low-dose, non-toxic chemotherapeutic would be critical to ascertain if the MIC-A/B induction will sensitize cancer cells to NK cells. In addition, as cancer cells evade NK cell recognition by shedding or cleavage of the MIC-A/B, it is imperative to determine whether low-dose chemotherapy mitigates or inhibits such shedding of MIC-A/B. Thus, current chemotherapeutics may have an extended application to induce or enhance cancer’s sensitivity to NK cell mediated cytotoxicity.

## Author Contributions

KQ and SG-K designed, discussed, and wrote the manuscript.

## Conflict of Interest Statement

The authors declare that the research was conducted in the absence of any commercial or financial relationships that could be construed as a potential conflict of interest.
